# Submergence Causes Similar Carbohydrate Starvation but Faster Post-Stress Recovery than Darkness in *Alternanthera philoxeroides* Plants

**DOI:** 10.1371/journal.pone.0165193

**Published:** 2016-10-24

**Authors:** Xiao qi Ye, Jin liu Meng, Bo Zeng, Ming Wu, Ye yi Zhang, Xiao ping Zhang

**Affiliations:** 1 Institute of Subtropical Forestry, Chinese Academy of Forestry/Research Station of Hangzhou Bay Wetlands Ecosystem, National Forestry Bureau, Fuyang, China; 2 Key Laboratory of Eco-environments in Three Gorges Reservoir Region (Ministry of Education), Chongqing Key Laboratory of Plant Ecology and Resources in Three Gorges Reservoir Region, School of Life Science, Southwest University, Chongqing, China; Universidade Federal de Viçosa, BRAZIL

## Abstract

Carbon assimilation by submerged plants is greatly reduced due to low light levels. It is hypothesized that submergence reduces carbohydrate contents and that plants recover from submergence in the same way as darkness-treated plants. To test this hypothesis, the responses of plants to submergence and darkness were studied and compared. Plants of a submergence-tolerant species, *Alternanthera philoxeroides*, were exposed to well drained and illuminated conditions, complete submergence conditions or darkness conditions followed by a recovery growth period in a controlled experiment. The biomass maintenance and accumulation, carbohydrate content dynamics and respiration rate in the plants were assessed to quantify the carbohydrate utilization rate and regrowth. The submerged plants maintained higher chlorophyll contents, more green leaf tissue and more biomass; recovered more quickly; and accumulated more carbohydrates and biomass than darkness-treated plants. The respiration rate was continuously reduced in the same pattern under both stress conditions but was maintained at a significantly lower level in the submerged plants; the total soluble sugar and total fructan contents were decreased at approximately the same rate of decrease, reaching similar low levels, in the two stress treatments. The *A*. *philoxeroides* plants were more tolerant of submergence than darkness. The faster recovery of desubmerged plants could not be explained by the similar carbohydrate contents at the start of recovery. Other types of carbon reserves besides carbohydrates or other mechanisms such as higher post-stress photosynthetic performance might be involved.

## Introduction

Complete submergence induces many stresses on terrestrial plant species [1], two of which are decreased light availability and low O_2_ and CO_2_ concentrations due to slow gas diffusion rates in the water [2–3]. The light availability under water is low due to the light reflection by the water surface and the light absorption by suspended particles, zooplankton, algae and silt [4–6]. Thus, aquatic environments are generally analogous to shaded environments in terms of the light climate [2], and the potential for underwater photosynthesis is consequently greatly reduced. Furthermore, the availability of the inorganic carbon supply for photosynthesis is severely limited [7–8] because of the 1/10,000 slower gas diffusion in water than in the air [9] and the boundary layers formed between the water column and submerged plant organs [10]. Submerged plants suffer from prolonged carbon starvation. As a major carbon reserve in plants, carbohydrates provide respiration and growth substrates for recovery from submergence. The dependence of post-stress recovery on carbohydrate availability was shown in plants experiencing stress events, such as defoliation due to grazing [11] or fire [12] and anoxia caused by ice cover [13]. In particular, the association between metabolism and submergence tolerance has been extensively studied in rice, a relatively flood-tolerant species [14–15]. In several rice varieties, submergence survival was positively associated with the carbohydrate levels prior to recovery [6, 16]. In wild wetland plant species, some studies have suggested that carbohydrates also play important roles in tolerance to flooding or flooding-induced, oxygen-deficient conditions. We propose that carbohydrate availability also plays an important role in the recovery growth from submergence.

The quantity of carbohydrates available for post-submergence recovery depends on the amount of carbohydrates consumed during the submergence period, which is largely by controlled by the oxygen availability. Under natural flooding conditions, such as in fast-moving river water, the oxygen availability may not limit carbohydrate utilization in submerged plants. Under such circumstances, submerged conditions resemble darkness with respect to similar inhibition of photosynthesis and similar reduction in carbohydrate reserves for post-stress recovery. For tolerant terrestrial species, submergence tolerance could be simplified to a tolerance of pure darkness. To examine this hypothesis, *Alternanthera philoxeroides* (alligator weed), an invasive plant species in many regions of the world, was selected as a case study species. This species is frequently found growing in riparian areas and is highly tolerant to long-term submergence [17–18]. To understand the mechanisms involved in high flood tolerance, the responses of the carbohydrate reserve and respiration rate during submergence or darkness treatment and during the subsequent recovery period were compared. Specifically, two questions were addressed. (1) Do *A*. *philoxeroides* plants utilize carbohydrates and recover from complete submergence and darkness at a similar rate? (2) If not, can the difference in recovery potential from the two stress conditions be explained by the difference in the maintained carbohydrate supply?

## Materials and Methods

### Plant materials and growth conditions

A single clone of *A*. *philoxeroides* was collected from the riverside of Jialing River, a tributary of the Yangtze River, in September 2007. No specific permissions were required for collecting *A*. *philoxeroides* plants at this location because the riparian area is state-owned; the sampling caused very little disturbance to the environment; and *A*. *philoxeroides*, an invasive species in China, is not a protected species. The clone was transported to and cultured in a condition-controlled greenhouse. New plants were propagated from this single clone and transplanted to pots (380 cm^3^) filled with one volume of potting soil and one volume of sand. The plants were grown for 14 d in a growth chamber under well-controlled growth conditions (air temperature of 20°C, photosynthetically active radiation (PAR) of 102 μmol photons · m^-2^ · s^-1^ and 16 hours of illumination per day). The light intensity was measured using a light sensor (X1_2_ optometer, Gigahertz-Optik GmbH, München, Germany).

### Submergence and darkness treatments

All treatments were conducted in the laboratory. Prior to treatment (day 0), some plants were harvested to determine the initial dry mass, carbohydrate levels (day 0, n = 5) and respiration rate (day 0, n = 5). The plants were exposed to (1) non-submergence with illumination (as a control, well drained and illuminated with PAR of 102 μmol photons · m^-2^ · s^-1^), (2) submergence (in compete darkness from the cover of a shade cloth) or (3) complete darkness without submergence. For the submergence treatment, the plants were immersed in water in six plastic containers (each containing 17 plants and 250 liters of water at an 80-cm depth). During the treatment period, the shoot length of the submerged plants increased from 18.8 ±1.1 cm (mean ± stand error) on day 0 to 56.3 ±3.8 cm on day 10, and the depth of the water above the plants was approximately 60 to 24 cm. The water in the containers was saturated with air (O_2_: 290 μmol · L^-1^; total inorganic carbon: 44.8 μmol · L^-1^; free CO_2_: 11.2 μmol · L^-1^) through the continuous gentle pumping of air into the water. The dissolved O_2_ was measured using a fiber-optic oxygen meter (Microx TX3, PreSens Precision Sensing GmbH, Regensburg, Germany), and the total inorganic CO_2_ was estimated using a titration method [19]. The pH of the tap water used was 8.2–8.4, and the water temperature was maintained at 20 ± 0.1°C throughout the entire treatment period. For the darkness treatments, all the plants were placed in a growth chamber (3 × 2 × 2 m) covered with four layers of black cloth. Air was circulated using an air pump. No light was detected in the chamber. Every other day (day 2, 4, 6, 8 and 10), some plants subjected to each treatment were harvested to measure the plant dry mass (n = 5), carbohydrate content (n = 5), and respiration rate (n = 5). On day 10, an additional five plants from each of the three treatments were harvested and divided into leaf, stem and root samples. The leaf chlorophyll content (n = 5) and the respiration rates of the leaves, stems and roots were measured (n = 5).

### Recovery from the submergence and darkness treatments

After ten days of submergence or darkness treatment, the remaining plants in each treatment were returned to non-submergence conditions, similar to the control plants. The plants were harvested after three and six days of recovery (day 13 and 16, respectively), and the respiration rate, dry mass and carbohydrate content were measured.

### Respiration rate measurements

The plants (or the tissues from the additional plants harvested on day 10) were rinsed with tap water, and the respiration rate was immediately measured. Respiration activity was estimated as the oxygen uptake rate of a single entire plant. The plants (tissues) were enclosed in airtight glass bottles completely filled with 1.1 L of tap water. The water was saturated with air prior to measurement (O_2_: 290 μmol · L^-1^; total inorganic carbon: 44.8 μmol · L^-1^; free CO_2_: 11.2 μmol · L^-1^) and had a constant temperature of 20 ± 0.1°C. The oxygen sensor (the same type as above) was inserted into the bottle through a rubber stopper. The system was assessed for leakage prior to the measurements. The incubation water was stirred using a magnetic stirrer to homogenize the oxygen concentration. The oxygen concentration in the bottle was monitored using a sensor connected to a datalogger. The incubation time was 20–30 min. This incubation was short enough that the respiration rate was not limited by the decreasing O_2_ concentration. The pH of the water decreased during the incubation, but the decrease was less than 0.3. The respiration rate of the entire plant or any tissue was calculated as

$$\text{Oxygen uptake rate }=\;\frac{\left(C_{\text{O2}\left(0\right)}-\;C_{\text{O2}(\text{t})}\right)\times V}{t\times DM}$$

*C*_O2(0)_: initial O_2_ concentration (mg /L) in the water (just before incubation)

*t*: incubation duration (hour)

*C*_O2(*t*)_: final O_2_ concentration (mg/L) in the water (just after incubation for a period time of t)

*V*: water volume (L)

*DM*: dry mass (g) of the whole plant or tissue

### Carbohydrate measurements

The harvested plants were quickly freeze-dried, and the different organs (leaves, stems and roots) were weighed and ground into powder. The glucose, fructose and sucrose contents in the organs were measured according to the enzymatic method [20]. The starch contents in the organs were assessed by a method using amyloglucosidase and α-amylase (Roche Diagnostics GmbH, Mannheim, Germany). Starch was not detected in these organs, indicating an absence of starch in this species. Therefore, starch was not measured in the experiment. Next, another important polysaccharide, fructan, was measured using an enzymatic Fructan Assay Kit (Megazyme Ltd., I Wicklow, reland), and a high amount of fructans, instead of starch, was observed in the plants. Thus, the fructan content in various plant parts was analyzed using the same Fructan Assay Kit.

### Chlorophyll measurements

The chlorophyll in the first batch of mature leaves was extracted with 96% ethanol and then spectrophotometrically determined (DR 5000 spectrophotometer, Hach Lange GmbH, Germany). The equation of Wintermans & de Mots [21] was used to calculate the concentration of chlorophyll *a* and *b*.

### Data analysis

All the statistical analyses were conducted using SigmaStat 2.03 (SPSS Inc., Chicago). The effects of the treatment duration and type (control, submergence and darkness) on the carbohydrate contents (total sugars and total fructans) were statistically explored using two-way ANOVA or Kruskal-Wallis test. When necessary, the effects of the duration within each treatment or of the treatment type within each sampling date were evaluated using one-way ANOVA. Multiple comparisons between the treatments were made to detect differences between the submergence and darkness treatments. The effects of the treatment type on the leaf chlorophyll content, green leaf biomass and total plant biomass were analyzed using one-way ANOVA or Kruskal-Wallis test, and multiple comparisons between the treatments were made.

## Results

### Biomass and leaf chlorophyll content

The plant biomass was determined on days 10 and 16 and compared among the 3 treatments (Table 1). The plant biomass and total green leaf biomass of individual plants significantly increased after only 6 days of recovery from both submergence and darkness treatments (P < 0.05). The submerged plants maintained more plant and leaf biomass than the darkness-treated plants on day 10 prior to recovery (Table 1). On day 16, the plant biomass and total green leaf biomass were much higher in the plants that had recovered from submergence compared with those recovered from the darkness treatment.

**Table 1:** Chlorophyll contents (mg · g^-1^ fresh leaf mass) on day 10 (after 10 days of submergence or darkness treatment), and the total green leaf biomass and plant biomass of *A*. *philoxeroides* plants (dry weight, g) on days 10 and 16 (after 10 days of submergence or darkness treatment and an additional 6 days of recovery). The data are presented as the means ± SE, *n = 5*. Different letters (a, b and c) indicate significant differences within each parameter on each sampling day (P<0.05); the same letters indicate no differences within each parameter on each sampling day (P>0.05).

		Control	Submergence	Darkness
*Chl a*	*Day 10*	0.74^b^	0.89^a^	0.61^c^
*Chl b*	*Day 10*	0.22^b^	0.27^a^	0.18^b^
Total green Leaf biomass	*Day 10*	0.34^a^	0.16^b^	0.11^c^
	*Day 16*	0.54^a^	0.26^b^	0.17^c^
Plant biomass	*Day* 10	0.92^a^	0.35^b^	0.30^b^
	*Day* 16	1.66^a^	0.60^b^	0.41^c^

A large proportion of leaves (older leaves) were senescent during the 10 days of darkness treatment, whereas no leaf senescence occurred under submergence treatment. On day 10, the chlorophyll content was determined from the first batch of fully grown leaves on the plants and compared among the 3 treatments (Table 1). The contents of both *Chl a* and *Chl b* were higher in the submerged plants and lower in the darkness-treated plants, compared with the control plants, and the difference between the submergence and darkness treatments was significant (Table 1, P < 0.05).

### Carbohydrates

**Fig 1:**
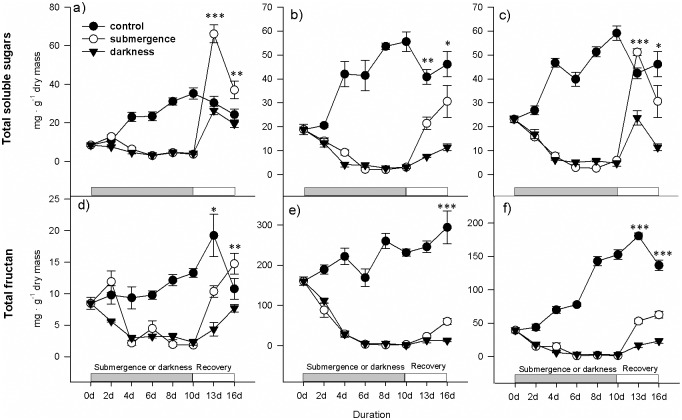
Total soluble sugars (glucose + fructose + sucrose, mg · g^-1^ dry mass) and total fructan (mg · g^-1^ dry mass) contents in *A*. *philoxeroides* plants. The upper row shows the total soluble sugars content; the lower row shows the total fructan content. Different tissues were separately investigated: a and d for leaves, b and e for stems, and c and f for roots. The plants were exposed to three different treatments for 10 days (day 0-day 10): 1) well drained and illuminated (control, black and closed circles), 2) submergence in darkness (white and open circles) or 3) non-submergence in darkness (triangles). From day 10 on, the plants were exposed to the normal growth conditions experienced by the control plants. The data are presented as the means ± SE, n = 5. For clarity, the statistical significance is only shown for the differences between the submergence/darkness and non-submergence/darkness treatments at each sampling time. *, P<0.05; **, P < 0.01; *** P < 0.001.

In *A*. *philoxeroides* plants, fructans were the major carbohydrate reserve, while soluble sugars made up only a small portion of the total carbohydrate pool (Fig 1). A large amount of fructans (150–300 mg · g^-1^ dry mass in stems and 50–200 mg · g^-1^ dry mass in roots, Fig 1e and 1f) was detected in the control plants (non-submergence and full illumination). For the control treatment, the total soluble sugars and fructan contents increased during days 0–10, reaching the highest levels on days 10–16, with similar patterns observed in the leaves, stems and roots (Fig 1).

The submergence and darkness treatments markedly impacted the carbohydrate content. On day 2, the total soluble sugars and fructan contents in the leaves of the submerged plants increased compared with those on day 0; however, these differences were insignificant (P < 0.05). In the leaves of the darkness-treated plants, the total soluble sugars content significantly decreased (P < 0.05), and the total fructan content insignificantly decreased (P > 0.05) (Fig 1a and 1d). Subsequently, the total soluble sugars and total fructan contents in all tissues continuously decreased until day 6, when these parameters were significantly lower compared with the corresponding values on day 0 (Fig 1, P < 0.05). From days 6–10, the carbohydrate content was relatively stable, indicating a minimum level in the tissues (Fig 1, P > 0.05). There were no significant differences in the total soluble sugars content between the submerged and darkness-treated plants during days 2–10 (P > 0.05), with the exception of higher total soluble sugars in the leaves of the submerged plants than in leaves of the darkness-treated plants on day 2 (Fig 1a).

The submerged and darkness-treated plants recovered under normal growth conditions after 10 days of treatment. After 3 days of recovery, on day 13, both the total soluble sugars and total fructan contents strongly increased in all tissues in both stress treatments, except for the fructan content (P <0.05) in the stems (Fig 1). From days 13–16, the total soluble sugars content continued to increase in the stems (Fig 1b) but decreased in the leaves and roots (Fig 1a and 1c). Both the total soluble sugars and total fructan contents were significantly higher (P < 0.05) in the plants that had recovered from submergence compared with the plants that had recovered from darkness on both days 13 and 16, except for the fructan content in the stems on day 13 (Fig 1).

### Respiration

**Fig 2:**
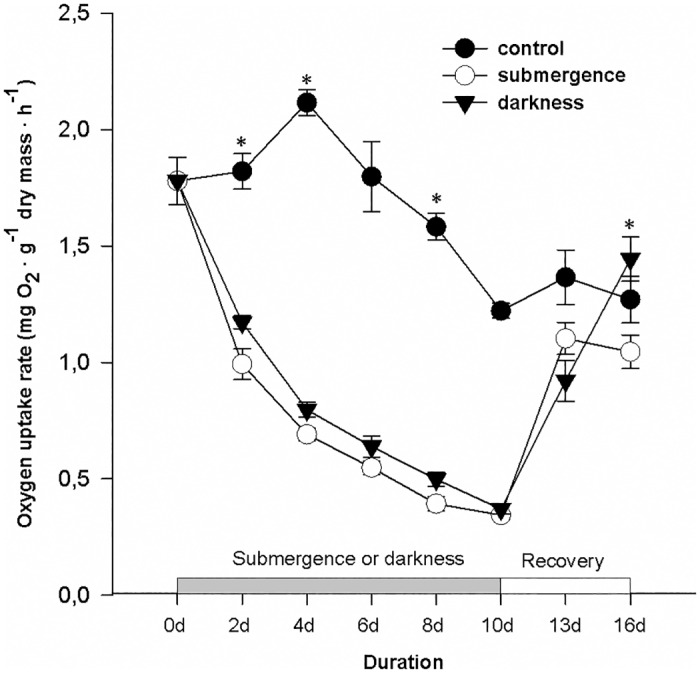
Respiration rate (mg O_2_ · g^-1^ dry mass · h^-1^) in entire individual *A*. *philoxeroides* plants. The plants were exposed to three different treatments for 10 days (0–10 d): 1) well drained and illuminated (control, black and closed circles), 2) submergence in darkness (white and open circles) or 3) non-submergence in darkness (triangles). From 10 d on, the plants were exposed to the normal growth conditions experienced by the control plants. The data are presented as the means ± SE, n = 5. For clarity, the statistical significance is shown only for the differences between the submergence/darkness and non-submergence/darkness at each sampling time. *, P < 0.05.

**Fig 3:**
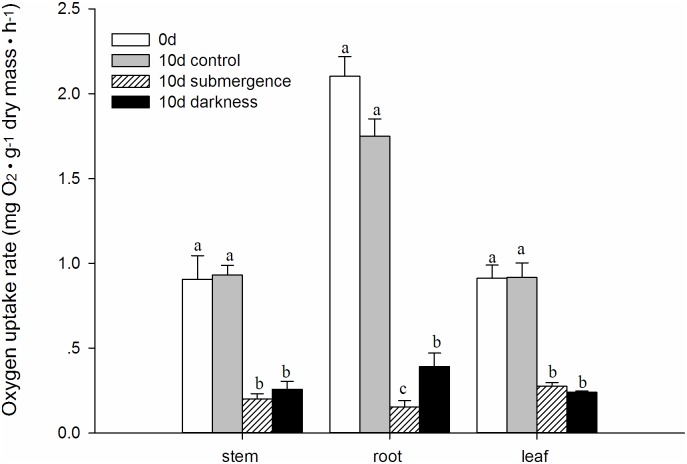
Respiration rate (mg O_2_ · g^-1^ dry mass · h^-1^) in different tissues (stem, root and leaf) of *A*. *philoxeroides* plants subjected to various treatments. The respiration rates were measured on day 0 prior to the initiation of the treatments (white and open columns) and on day 10. Three treatments were performed in this study: (1) well drained and illuminated (gray and closed columns), (2) submergence in darkness (gray and crossed columns) and (c) non-submergence in darkness (black and closed columns). The total amount of oxygen uptake was recorded and calculated on a dry mass basis in each tissue. The data are presented as the means ± SE, n = 5. Different letters indicate significant differences within each tissue (P < 0.05), and the same letters indicate no differences within each tissue (P > 0.05).

The sampling time for the respiration measurements was the same as that for the carbohydrate content analysis. The submergence and darkness treatments exerted strong effects on the dry mass-normalized respiration rate of entire individual plants (Fig 2). In the control plants, respiration increased during days 0–4 but started to decrease from day 6 until day 13. Despite this decrease, the respiration rate in control plants was consistently higher than that in submerged or darkness-treated plants during the treatment period (days 2–10, P < 0.05). Both the submergence and darkness treatments strongly reduced the respiration rate on day 2, and the rate continued to decrease during days 2–10 to extremely low levels (approximately 0.4 mg O_2_ · g dry mass · h^-1^). Despite the similar reduction and decrease in respiration rate, the respiration rate in the submerged plants was higher than that in the darkness-treated plants. This difference was significant on days 2, 4 and 6 (P > 0.05). Subsequently, as recovery was initiated, the respiration rates in both the submerged and darkness-treated plants were markedly enhanced from days 10–13, and these rates remained almost the same on day 16 in formerly submerged plants and continued to increase in formerly darkness-treated plants. Additionally, the respiration rates in the leaves, stems and roots were investigated separately on day 10 (Fig 3). The tissue-specific respiration rates in the submerged and darkness-treated plants were compared. Similar to the respiration rates in entire individual plants, the respiration rates in the stems, roots and leaves were markedly reduced compared with the respiration rates recorded on day 0 and in the control plants on day 10. On day 10, respiration in the stems or leaves was not significantly different (Fig 3) P < 0.05) between the submergence and darkness treatments, but respiration in the roots was much higher in the submerged plants (Fig 3, P < 0.05).

## Discussion

It has been hypothesized that *A*. *philoxeroides* plants would recover at similar rates from complete submergence and darkness treatments. However, in our study, submerged *A*. *philoxeroides* plants recovered much faster than darkness-treated plants, gaining more biomass (Table 1) and accumulating more carbohydrates (Fig 1), suggesting that the darkness treatment was more stressful than the submergence treatment. Our results are contrary to the findings of Luo *et al*. [22], who reported that submerged plants (in darkness) recovered more slowly than shaded plants (without submergence). The cause of these different results could reflect the different experimental setup. In our study, the complete darkness treatment prevented any illumination; thus, photosynthesis did not occur, and the plants were completely carbon-starved. Furthermore, in the present study, the concentration of oxygen in the water was much higher (296 μmol/L, air-saturated at 20°C) than in the submergence treatments (151–225 μmol/L) of Luo *et al*. [22], and the transfer from the later low-oxygen conditions to de-submergence conditions caused a burst of reactive oxygen species and other toxic compounds, such as acetaldehyde, which may cause injuries [23].

We predicted that submerged and darkness-treated plants would show similar aspects of carbohydrate mobilization. Indeed, the submergence and darkness treatments similarly lowered the carbohydrate levels (Fig 1), and carbohydrate reserves were nearly depleted in both treatments just prior to recovery (Fig 1). We hypothesized that the carbohydrate reserves at the beginning of the post-submergence period plays an important role in recovery and that differences in carbohydrate availability prior to recovery could explain the differences in recovery from submergence and darkness. However, because the carbohydrate availability was equally diminished in submerged and darkness-treated plants (Fig 1), the difference in recovery from submergence or darkness cannot be attributed to the quantity of remaining carbohydrate reserves in the plants under the stress conditions in the present study. In plants, carbohydrate metabolism largely depends on the oxygen supply; therefore, carbohydrate mobilization is greatly influenced by the aeration conditions [13, 24–25], and the comparable carbohydrate contents in the stressed plants suggest that no oxygen limitation occurred for carbohydrate catabolism in the submerged or darkness-treated plants.

However, why did the submerged plants recover at a much faster rate than the darkness-treated plants? One probable explanation is that other types of carbon reserves than carbohydrates might contribute to the recovery processes when the stress duration is prolonged. This idea was supported by the fact that the respiration rate was higher in the submerged plants but the reduction in the carbohydrate content was similar in the two treatments (Figs 1 and 2). Both submergence and darkness could effectively downregulate metabolism, as reflected by the strongly reduced respiration rate in the entire plants or specific tissues (Figs 2 and 3). The respiration rate could be controlled by the availability of substrates. The higher respiration rate in darkness-treated plants suggests more abundant substrates for catabolic respiration. The discrepancy in the respiration rates and carbohydrate contents suggests a contribution to respiration from catabolic metabolites other than carbohydrates alone. This idea was further supported by the fact that most sugars and fructans were nearly depleted (Fig 1) after 4 days in the two stress treatments, but respiration continued (Figs 2 and 3), indicating that respiration could be predominately maintained by the catabolism of other metabolites, such as proteins, in this later stage [26].

Another possibility is that recovery from submergence might depend more on post-stress photosynthetic performance and less on stored carbon reserves. In *A*. *philoxeroides* plants, photosynthetic acclimation is important for the post-submergence recovery of photosynthesis and growth [22,27]. This idea was further supported by the results obtained in the present study. More functional green leaves were maintained in the submerged plants compared with the darkness-treated plants (Table 1), favoring more rapid carbon assimilation and accumulation at the whole-plant level. Furthermore, the capacity to maintain the integrity of the photosynthetic apparatus under stress conditions could play an important role. Although the photosynthetic capacity was not examined in the present study, the high chlorophyll contents in the submerged plant leaves (Table 1) could serve as an indicator that of well-maintained photosynthesis. Sarkar *et al*. [28] showed that tolerant rice cultivars could maintain higher chlorophyll contents than intolerant cultivars. In a carbon starvation-tolerant species, the pearl millet, chloroplasts and other ultrastructures were maintained after 8 days in darkness, despite high rates of carbohydrate degradation [26].

The fast recovery from submergence and darkness stress conditions might shed light on the aggressiveness of *A*. *philoxeroides* plants in both terrestrial and aquatic habitats [29–30]. However, the mechanisms behind this tolerance should be intensively examined in future studies. Examining how the concurrence of darkness exerts further stress on submerged plants will help to compare the differential responses to submergence and darkness and provide a promising approach to achieve a better understanding of submergence. In conclusion, the submerged *A*. *philoxeroides* plants recovered faster than the darkness-treated plants, and the difference in recovery growth was not due to differing carbohydrate availability prior to recovery.

## Supporting Information

S1 Data S1 Data for Fig 1. Total soluble sugars (glucose + fructose + sucrose, mg · g^-1^ dry mass) and total fructan (mg · g^-1^ dry mass) contents in *A*. *philoxeroides* plants.
(XLS)

S2 Data S2 Data for Fig 2. Respiration rate (mg O_2_ · g^-1^ dry mass · h^-1^, data are the means ± SE, n = 5) in entire individual *A*. *philoxeroides* plants.
(XLS)

S3 Data S3 Data for Fig 3. Respiration rate (mg O_2_ · g^-1^ dry mass · h^-1^) in different tissues (stem, root and leaf) of *A*. *philoxeroides* plants subjected to various treatments.
(XLS)
